# Possibility of sandwiched liver surgery with molecular targeting drugs, cetuximab and bevacizumab on colon cancer liver metastases: a case report

**DOI:** 10.1186/1477-7819-10-129

**Published:** 2012-06-29

**Authors:** Yoichi Toyama, Takuro Ushigome, Kazuhiro Watanabe, Hiroaki Kitamura, Shinji Onda, Ryota Saito, Seiya Yoshida, Hidejiro Kawahara, Satoru Yanagisawa, Katsuhiko Yanaga

**Affiliations:** 1 Department of Surgery, The Jikei University Kashiwa Hospital, 163-1, Kashiwashita, Chiba Prefecture, Kashiwa City 277-8567, Japan; 2 Department of Surgery, The Jikei University School of Medicine, 3-25-8, , Nishishinnbashi, Minato ku, Tokyo, 105-8461, Japan; 3 Department of Surgery, Jikei University Kashiwa Hospital, 163-1, Kashiwashita, Chiba Prefecture, Kashiwa City 277-8567, Japan

**Keywords:** Bevacizumab, Cetuximab, Colon cancer liver metastases, Molecular targeting drug, Sandwiched liver surgery

## Abstract

A 31-year-old man with sigmoid colon cancer with concomitant simultaneous multiple liver metastases had received FOLFIRI (leucovorin, fluorouracil and irinotecan) and FOLFOX6 (leucovorin, fluorouracil and oxaliplatin) after an ordinary sigmoidectomy. However, his serum carcinoembryonic antigen (CEA) level increased rapidly during the fifteen months after the operation while he was on FOLFOX6. Abdominal computed tomography revealed expanding multiple liver tumors. As the third line chemotherapy, a combination therapy of cetuximab with irinotecan was given, which markedly reduced his levels of serum CEA, and the size and number of liver tumors. He underwent lateral segmentectomy of the liver and microwave coagulation of the liver metastases in the remnant liver. Thereafter, a good quality of life with tumor dormancy was obtained for 6 months. However, his serum CEA started to rise again in the absence of liver tumors. Therefore, FOLFOX6 with bevacizumab was chosen as the fourth line chemotherapy, and the serum CEA was reduced with tumor dormancy. A good quality of life was obtained again at 3 years after the first surgery. This report indicates the effectiveness of sandwiched liver surgery with the molecular targeting drugs cetuximab and bevacizumab on multiple liver metastases of colon cancer, and suggests the possibility of a regimen consisting of bevacizumab following cetuximab.

## Background

Recently, various molecular targeting drugs have appeared through developing biotechnology [1]. Cetuximab, a new molecular drug, has a notable ability as an anti-epidermal growth factor receptor (EGFR) monoclonal antibody [2]. A randomized European study suggested that cetuximab was effective in patients with irinotecan-refractory metastatic colorectal cancer [3]. A *KRAS* mutation is an important predictive factor for resistance to cetuximab chemotherapy in patients with metastatic colorectal cancer [4]. Moreover, it has been reported that the combination of cetuximab and chemotherapy improves the resectability of colorectal cancer liver metastases (CCLM) [5]. Bevacizumab, an anti-vascular endothelial growth factor receptor (VEGFR) monoclonal antibody, is also an important drug among these new agents [6]. An open-label study, NO16966, reported the non-inferiority of XELOX (capecitabine and oxaliplatin) to FOLFOX4 (leucovorin (LV), fluorouracil and oxaliplatin) for the first line treatment of metastatic colorectal cancer; however, the additive effect of bevacizumab to the two chemotherapies was not ultimately observed [7-9]. However, the addition of bevacizumab to FOLFOX4 was effective in metastatic colorectal cancer, including in patients with CCLM after first line chemotherapy with FOLFIRI (LV, fluorouracil and irinotecan) [10]. Furthermore, some reports have indicated that bevacizumab is effective in advanced colorectal cancer refractory to irinotecan, oxaliplatin or cetuximab [11-14]. We herein report a young male patient with CCLM who was treated successfully by a timely sandwiched liver surgery with the molecular targeting drugs, cetuximab and bevacizumab after treatment with FOLFIRI and FOLFOX regimens.

## Case presentation

A 31-year-old man complained of melena and underwent a colonoscopy that identified a two-thirds circumferential type 2 tumor, an advanced sigmoid cancer. Abdominal computed tomography (CT) showed numerous CCLM. The patient underwent a sigmoidectomy with standard lymph node dissection in our department and histopathological findings revealed a moderately differentiated adenocarcinoma.

**Figure 1:**
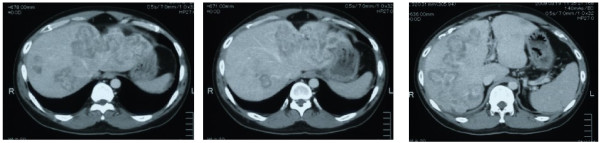
Computed tomography demonstrated expanding multiple liver metastases of the sigmoid colon cancer after FOLFIRI and FOLFOX6 therapy.

**Figure 2:**
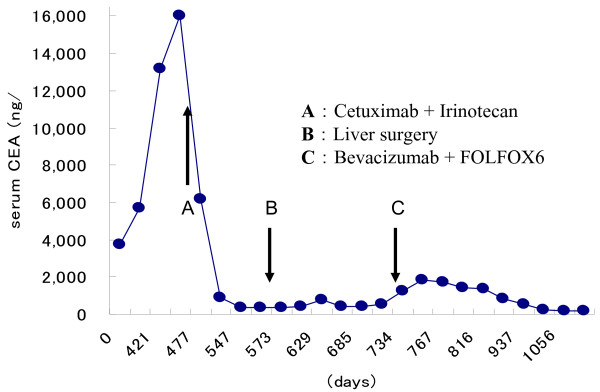
Changes in the serum carcinoembryonic antigen levels of the patient with colon liver metastases after sigmoidectomy.

**Figure 3:**
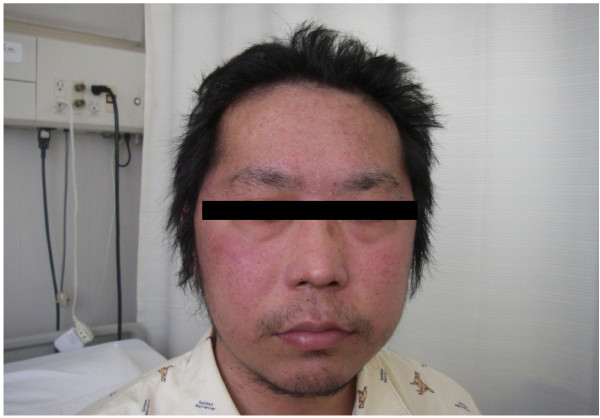
Face eczema appeared as a principal side effect of cetuximab.

**Figure 4:**
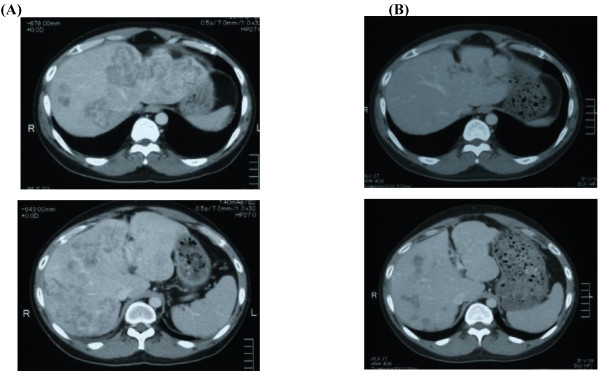
**Reduction of tumor volume in the liver after the cetuximab and irinotecan treatment.** (**a**) Computed tomography before and (**b**) after the combination treatment with cetuximab and irinotecan.

The patient underwent conventional neoadjuvant chemotherapy, first with FOLFIRI (5-fluorocil (FU) 400 mg/m^2^ bolus injection; LV 400 mg/m^2^/2 hours; 5FU 2,400 to 3,000 mg/m^2^/46 hours continuous infusion with irinotecan 180 mg/m^2^/1.5 hours, every 2 weeks for twenty courses). He was then commenced on FOLFOX6 (Day 1: 5FU 400 mg/m^2^ bolus injection; LV 200 mg/m^2^/2 hours; 5FU 600 mg/m^2^/22 hours continuous infusion with oxaliplatin (L-OHP) 85 mg/m^2^/2 hours; Day 2: same menu without L-OHP, every 2 weeks for eight courses) because abdominal enhanced CT demonstrated enlargement of the CCLM according to Response Evaluation Criteria in Solid Tumors (RECIST) (Figure 1). However, in spite of the intensive neoadjuvant chemotherapies, his serum carcinoembryonic antigen (CEA) level gradually increased during the fifteen months following the first operation (Figure 2). Since the cancer cells were found to have wild type *KRAS*, a combination therapy of cetuximab with irinotecan was chosen as the third line chemotherapy, considering the possibility of liver surgery for the CCLM. The patient received weekly cetuximab (400 mg/m^2^ initial dose and 250 mg/m^2^ per week thereafter) and bi-weekly irinotecan (150 mg/m^2^). One course of the combination therapy was defined as 7 weeks (six administrations of cetuximab and three administrations of irinotecan, followed by one week’s rest). Although the patient initially suffered from facial eczema due to the cetuximab (Figure 3), follow-up abdominal enhanced CT demonstrated a marked reduction in the size and number of CCLM (Figure 4), and the serum CEA decreased significantly (Figure 2).

**Figure 5:**
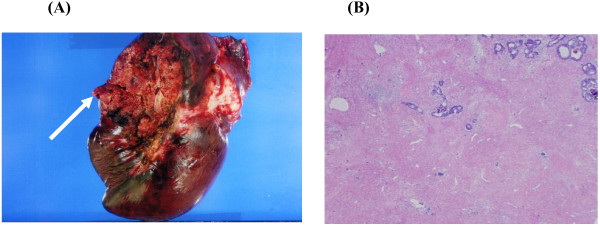
**Surgical specimen and pathological findings of the second operation.** (**a**) The arrow indicated the cut face side of extirpated lateral segment of the liver. (**b**) Hemotoxylin and eosin staining (× 40) showing a moderately differentiated adenocarcinoma.

**Figure 6:**
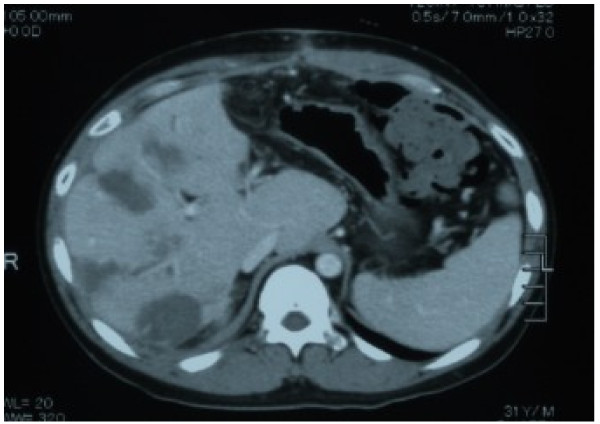
Computed tomography showed multiple low-density areas in the remnant liver following microwave coagulation.

To obtain further tumor reduction, the patient underwent lateral segmentectomy of the liver (Figure 5a) and microwave coagulation for multiple CCLM in the remnant liver. At the second-look operation in this case, obvious hepatotoxicities as such as steatohepatitis and blue liver phenomenon were observed macroscopically. Histology of the operative specimen showed a poorly differentiated metastatic adenocarcinoma derived from colon cancer (Figure 5b). As shown in Figure 6, postoperative findings by abdominal enhanced CT displayed no variable lesions in the remnant liver.

The patient obtained good quality of life (QOL) with tumor dormancy by addition of the third line chemotherapy during the 6 months after the second operation. However, his serum CEA level gradually re-increased even though the third line chemotherapy with the combination of cetuximab and irinotecan was performed (Figure 2). Accordingly, a combination of FOLFOX6 with bevacizumab was chosen as the fourth line chemotherapy. His serum CEA level again decreased significantly without any new lesions in the remnant liver. The patient has kept a good QOL with tumor dormancy as of 3 years after the first operation.

## Discussion

Cetuximab, a new molecular drug with an extracellular action site, is expected to remarkably suppress tumor cell mitosis as an anti-EGFR monoclonal antibody [2]. Additionally, it is known that bevacizumab, a new monoclonal antibody against VEGFR, also inhibits tumor cell proliferation significantly by acting on extracellular receptor sites [6]. A European randomized trial suggested that cetuximab with or without irinotecan was effective in patients with irinotecan-refractory CCLM [3]. Thereafter, cetuximab combined with chemotherapies has led to a better prognosis, especially in *KRAS* wild type patients with CCLM [4]. This was supported by the National Cancer Institute of Canada Clinical Trials Group and Australasian Gastro-Intestinal Trials Group CO.17 trial, which demonstrated that cetuximab offers good QOL and survival benefits for pretreated patients with advanced, wild-type *KRAS* colorectal cancer [15]. A European Organisation for Research and Treatment of Cancer trial demonstrated that perioperative FOLFOX4 chemotherapy with surgery had advantages over surgery alone [16]. Thus, to obtain prolonged survival of patients with CCLM, reduction surgery may be effective. Adam *et al*. reported that cetuximab rescued some patients with CCLM by increasing the resectability of those tumors [5]. The CELIM study by European groups suggested that neoadjuvant chemotherapy with cetuximab yielded high response rates and significantly increased resectability when compared with historical controls [17]. A European Prospective Investigation into Cancer and Nutrition study indicated that cetuximab with irinotecan improved the QOL in patients with CCLM who failed to exhibit a good response with fluoropyrimidine and oxaliplatin therapy [18]. Based on these previous studies, we chose the combination therapy of cetuximab with irinotecan as the third line chemotherapy for our patient with the wild type *KRAS* gene. Consequently, the patient could undergo liver surgery and obtain a good QOL with a significant reduction in his serum CEA level over the next 6 months.

Some chemotherapeutic agents have been reported to elicit hepatotoxicities, for example, irinotecan associated with steatohepatitis [19]. Oxaliplatin has also induced toxic liver injury, which manifests as sinusoidal dilatation or sinusoidal obstruction syndrome, namely blue liver, and nodular regenerative hyperplasia [20]. However, Pessaux *et al*. have suggested that popular chemotherapy with cetuximab or bevacizumab is not associated with definitive hepatotoxicities [21-23]. A recent study indicated that bevacizumab suppresses oxaliplatin-induced liver damage [24,25]. Although our patient’s liver demonstrated an abnormal gross appearance, there was no major impediment to performing the liver surgery safely.

Bevacizumab usually has been administrated with FOLFIRI or FOLFOX as first line chemotherapy in patients with CCLM and a poor prognosis, in the hope of obtaining its additive effect [7-9]. However, some reports have shown usefulness of bevacizumab-containing therapy for advanced colorectal cancer patients after failure of irinotecan, oxaliplatin and cetuximab [10-13]. Results from these reports led us to choose a combination of FOLFOX6 plus bevacizumab as the fourth line chemotherapy.

## Conclusion

We here describe the successful management of a young adult patient with CCLM with a liver surgery sandwiched between treatment with cetuximab and bevacizumab, suggesting the possibility of bevacizumab administration after cetuximab as a unique and effective therapeutic modality.

## Consent

Written informed consent was obtained from the patient for publication of this case report and accompanying images. A copy of the written consent is available for review by the Editor-in-Chief of this journal.

## Abbreviations

CCLM: colon cancer liver metastases; EGFR: epidermal growth factor receptor; FOLFIRI: leucovorin fluorouracil and irinotecan; FOLFOX: leucovorin fluorouracil and oxaliplatin; LV: Leucovorin; QOL: quality of life; RECIST: Response Evaluation Criteria in Solid Tumors; VGEFR: vascular endothelial growth factor receptor; XELOX: capecitabine and oxaliplatin.

## Competing interests

The authors declare that they have no competing interests.

## Authors’ contributions

YT and TU performed operation. YT, TU, KW and HK contributed to the conception of chemotherapies, including molecular targeting drugs. All authors analyzed and interpreted the patient data regarding its oncological features, and have been involved in drafting the manuscript. KY had given final approval of the version to be published. All authors read and approved the final manuscript.

## Authors’ information

The first author, Yoichi Toyama is specialized in hepatobiliary pancreatic surgery, laparoscopic hepatectomy and pancreatectomy in particular. The second author, Takuro Ushigome, is an expert in colorectal surgery and chemotherapy.
